# Exercise Prescription Intervention Rehabilitation Suggestions for Fatty Liver Patients

**DOI:** 10.1155/2022/2506327

**Published:** 2022-04-16

**Authors:** Tian Wan, Kun-Da Hong, Si-Yu Lu

**Affiliations:** Department of Rehabilitation Medicine, The Second Affiliated Hospital of Fujian Medical University, Quanzhou, Fujian Province, China

## Abstract

In this study, the exercise prescription intervention rehabilitation suggestions for fatty liver patients were summarized as follows: first, basic exercises (brisk walking and jogging.), sports (swimming, badminton, and cycling), traditional Chinese medicine exercises (Taichi boxing and eight-section brocade), the aim of which is to improve overall physical strength and endurance of the body; second, exercise intensity, duration, and frequency; third, exercise precautions; and fourth, exercise prescription selection and suggestion.

## 1. Introduction

Fatty liver, a common liver disease in the middle-aged and elderly, refers to the pathological changes of excessive fat accumulation in hepatocytes caused by various factors. In the clinical setting, the incidence of fatty liver is increasing year-after-year with a trend towards younger age of reference. There are no specific drugs or treatment for fatty liver at this time. According to numerous references, fatty liver can be reversed, especially when the patients adhere to a proper exercise program, dietary recommendations, and/or appropriate medications [1]. It has been reported that adopting exercise therapy in an effort to ultimately stop medication may be a more scientific and ideal way to treat fatty liver [2, 3]. In the current study, an exercise prescription was initiated to probe into the rehabilitation concept of exercise prescription intervention for fatty liver patients, and suggestions on standardized rehabilitation were advanced, which are summarized as follows.

## 2. Exercise Items

The exercise prescription items included the following: aerobic exercises aimed at improving overall physical strength and endurance, such as brisk walking, jogging, badminton, cycling, Taichi boxing, and eight-section brocade.

### 2.1. Basic Exercises: Brisk Walking and Jogging

Brisk walking and jogging are currently the most popular exercises and have the advantages of easily controlled exercise intensity and volume, fewer sports injuries, and simple and easy realization, which is suitable for various populations. Brisk walking is a type of low-intensity aerobic exercise, the essentials of which are as follows: first, breathe naturally and relax the body; second, keep the head up, chest out, and abdomen in; and third, swing the arms naturally and place the center of gravity on the feet. Jogging, also known as slow-paced running, is a type of medium-intensity aerobic exercise, the essentials of which are as follows: first, breathe naturally, relax the upper limbs, and keep the muscles of the lower limbs elastic to avoid injuries; second, lean forward, relax the shoulders, and swing the arms naturally (the range should be natural and comfortable); third, land gently on the forefeet.

References have revealed that brisk walking and jogging promote fat consumption to realize fast weight loss. Brisk walking and jogging can help maintain cardiac function in the middle-aged and elderly, slow the decline in lung elasticity, and exert positive effects in preventing diseases, such as coronary heart disease, hypertension, and arteriosclerosis [4].

### 2.2. Sports: Swimming, Badminton, and Cycling

Because fatty liver currently has a younger age of onset than in the past, young people can achieve quick therapeutic efficacy through sports, such as swimming, badminton, and cycling, which have the advantages of high exercise intensity and fast benefits. Experimental research has confirmed that [5, 6] sports, by regulating the lipid metabolism, antioxidation, and inflammation suppression, may ameliorate the level of blood lipids in hyperlipidemic rats. The possible mechanisms underlying swimming in treating fatty liver have been reported as follows: first, sports can repress lipid synthesis in the liver by downregulating the levels of lipid synthesis-related gene expression, such as SREBP-1c and SCD1 [7–9]; second, sports can facilitate the phosphorylation of Akt in the liver and raise the sensitivity to insulin [10–12]; third, sports can upregulate the expression of PPAR*α*, thus enhancing the oxidation of fatty acids in the liver [13–15]. There are many references involving the treatment of fatty liver by swimming, but few studies on other sports are available. Nevertheless, badminton, cycling, and other aerobic exercises can also achieve good therapeutic efficacy on fatty liver.

### 2.3. Traditional Chinese Medicine (TCM) Exercises: Taichi Boxing and Eight-Section Brocade

Taichi boxing is in accordance with the principle of yin-yang change. Yin and yang interconvert into and complement each other in each move and each gesture. For this reason, Taichi boxing can recuperate yin and yang qi and blood of the entire body, soothe the liver by regulating qi, and reconcile yin and yang (Table 1).

Eight-section brocade, a regime invented by ancient Chinese medical specialists, is a type of TCM physical and breathing exercise, which is characterized by promoting the circulation of vital energy of human blood and restoring the health of human body through physical movements, such as pitching, stooping, and flexion and extension of limbs (Table 2).

**Figure 1:**
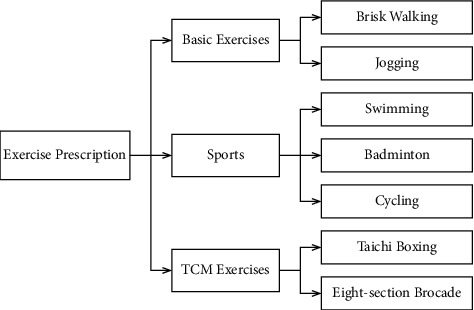
Exercise prescription.

Both Taichi boxing and eight-section brocade require cooperation with breathing, that is, exhalation through the mouth and inhalation through the nose. By expelling foul air through the mouth and breathing fresh air through the nose, the stale is expelled and the fresh is imbibed. Through such exhalation and inhalation, visceral movement can be enhanced, visceral microcirculation ameliorated, and liver metabolic function promoted, thus boosting rehabilitation. Hence, Taichi boxing and eight-section brocade can promote flow of qi and blood, dredge meridians, soothe the liver and gallbladder, recuperate internal organs, regulate yin and yang, prolong life, and prevent and cure diseases. Taichi boxing and eight-section brocade are very beneficial to subhealth population and the elderly, as well as fatty liver patients. A randomized controlled trial of Taichi have evaluated the effects of Taichi in ordinary adults aged 19–70 years. This study may provide valuable data on the effects of Taichi on hypertension [16]. Previous studies have showed positive effects of bone mineral density on maintaining eight-section brocade and improving functional outcome [17, 18]. It can be used to decreasing bone loss and the rate of osteoporotic fracture of patients [19]. Modern medical studies have suggested that both Taichi boxing and eight-section brocade are medium and low-intensity aerobic exercises [20, 21], and long-term practice of Taichi boxing and eight-section brocade can effectively modulate the lipid metabolism, thus ameliorating blood lipid levels, and ultimately treating fatty liver [22, 23] (Figure 1).

## 3. Exercise Intensity, Duration, and Frequency

### 3.1. Exercise Intensity

Long-term medium and low-intensity general aerobic exercises are most suitable for fatty liver patients. Because the majority of fatty liver patients are accompanied by different degrees of hypertension, overload exercise may induce cardiovascular and cerebrovascular diseases, so it is of vital importance to control the exercise intensity. Fatty liver patients should adopt different exercise intensities according to age and constitution, and the following criteria can be used for evaluation: first, the suggested heart rate variation range is 45–60 beats/min before and after exercise; second, the heart rate in the quiet state is taken as the standard, and it is suggested that the heart rate should revert to the standard level 3–5 min after exercise.

### 3.2. Exercise Duration and Frequency

The recommended frequency of brisk walking and jogging is 3–6 times per week for 40–90 min (or 3–6 km) each time. Attention should be paid to speed control, and it is best to sweat slightly. When the patients are out of breath, they are engaged in anaerobic exercise. In addition, the patients are suggested to do one of the sports, such as swimming, badminton, and cycling (2-3 times per week for 30–60 min each time) without feeling obvious fatigue after the sports. It is also recommended to practice one of the TCM exercises, such as Taichi boxing and eight-section brocade, 30–6 times per week for 30–45 min (2-3 repeats) each time.

## 4. Exercise Precautions

When creating an exercise prescription, fatty liver patients should pay attention to the following: first, undergo a physical examination regularly, which provides a basis for exercise prescription and exercise intensity control. Second, take necessary medications and do not discontinue medications abruptly. Third, develop regular and appropriate living habits and adopt a scientific and reasonable diet; no smoking, drinking alcohol, or lavish meals; and sleep regularly and do not stay up late.

## 5. Selection and Suggestions on Exercise Prescription

**Figure 2:**
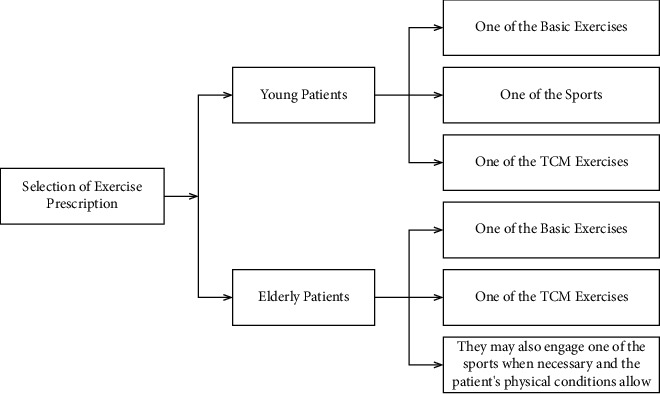
Selection of exercise prescription.

It is suggested in this study that young patients with fatty liver engage in one of the basic exercises, one of the sports, and one of the TCM exercises, while elderly patients who may have cardiovascular and cerebrovascular diseases adopt one of the basic exercises plus one of the TCM exercises (they may also engage in one of the sports when necessary and the patient's physical conditions allow). Exercise training for fatty liver patients should be done in an orderly way and step-by-step, and continuous and persistent training is the most important (Figure 2).

## 6. Summary

As an essential organ of the human lipid metabolism, the liver is mainly responsible for excretion of lipids into blood in the form of very low-density lipoprotein and synthesis of triglycerides by absorbing free fatty acids from blood [24]. At present, people are prone to overeating due to improved living standards. As a result, excessive calories are converted into fatty acids, which enter the liver beyond the processing capacity of the liver, thus accumulating in the liver and inducing fatty liver [25]. Thus far, no specific drug for fatty liver has been developed, and exercise training plays a positive and critical role in the treatment of fatty liver. In this study, therefore, the efficacy of different exercise trainings on fatty liver patients was investigated, and suggestions were proposed regarding exercise intensity, duration, frequency, and precautions. In addition, exercise prescriptions were advanced for patients of different ages. It is expected that with the help of more standardized exercise prescription suggestions, more scientific and reasonable exercise schemes can be provided for patients.

## Figures and Tables

**Table 1:** Specific operation of 24-style Taichi boxing.

Number	Procedure	Number	Procedure	Number	Procedure
1	Commencing form	9	Single whip	17	Push down and stand on one leg, right style
2	Part the wild horse's mane on both sides	10	Wave hands like clouds	18	Work at shuttles on both sides
3	White crane spreads its wings	11	Single whip	19	Needle at sea bottom
4	Brush knee and twist step on both sides	12	High pat on horse	20	Flash the arms
5	Play pipa	13	Kick with right heel	21	Turn, deflect downward, parry and punch
6	Repulse monkey on both sides	14	Strike opponent's ears with both fists	22	Apparent close up
7	Grasp the bird's tail, left side	15	Turn and kick with left heel	23	Cross hands
8	Grasp the bird's tail, right side	16	Push down and stand on one leg, left style	24	Closing form

**Table 2:** Specific operation of eight-section brocade.

Number	Procedure	Number	Procedure
1	Double hands push-up heaven	5	Thrusting the fists and making the eyes glare to enhance strength
2	Posing as an archer shooting both left- and right-handed	6	Moving the hands down the back and legs and touching the feet to strengthen the kidneys
3	Holding one arm aloft to regulate the functions of the spleen and stomach	7	Swinging the head and lowering the body to relieve stress
4	Looking backwards to prevent sickness and strain	8	Raising and lowering the heels to cure diseases

## Data Availability

The data used to support the findings of this study are available from the corresponding author upon request.
